# TALKING TIME: A pilot randomized controlled trial investigating social support for informal caregivers via the telephone

**DOI:** 10.1186/s12913-020-05523-9

**Published:** 2020-08-25

**Authors:** Martin Nikolaus Dichter, Bernd Albers, Diana Trutschel, Armin Michael Ströbel, Swantje Seismann-Petersen, Katharina Wermke, Margareta Halek, Martin Berwig

**Affiliations:** 1 German Center for Neurodegenerative Diseases (DZNE), Witten, Stockumer Straße 12, 58453 Witten, Germany; 2 School of Nursing Science, Witten/Herdecke University, Stockumer Straße 12, 58453 Witten, Germany; 3 Institute of Nursing Science, Medical Faculty, University of Cologne, Cologne, Germany; 4 Clinic and Policlinic for Psychiatry and Psychotherapy, Leipzig University, Semmelweisstraße 10, 04103 Leipzig, Germany

**Keywords:** Dementia, Informal caregivers, Psychosocial interventions, Telephone-based intervention, Social support, Health-related quality of life

## Abstract

**Background:**

Caring for people with dementia at home requires considerable time, organization and commitment. Therefore, informal caregivers of people with dementia are often overburdened. This study examined the effects of the telephone-based Talking Time intervention, which is an approach used to strengthen the psychological health-related quality of life (HRQoL) and social support of informal caregivers of people with dementia living at home.

**Methods:**

This study was a Medical Research Council framework phase two randomized controlled trial. The intervention consisted of a preliminary talk, information booklet, six structured telephone-based support group meetings and a structured written self-evaluation of each support group meeting. The control participants performed their usual individual self-organized care. After completing the data collection, the control group received the Talking Time intervention for fidelity reasons. The primary outcome was the self-rated psychological HRQoL of the informal caregivers, which was measured with the mental component summary of the General Health Survey Questionnaire Short Form 12 (SF-12).

**Results:**

Thirty-eight informal caregivers and their relatives were included and allocated to the intervention or control groups (*n* = 19 each). After 3 months, the Talking Time intervention group demonstrated an increase in the self-rated psychological HRQoL scores, whereas the scores decreased in the control group. However, the standardized effect size of 1.65 (95% Confidence Interval, − 0.44 – 3.75) was not significant. Additionally, the secondary outcomes demonstrated no significant results. The differences between the groups in most outcomes were in the expected direction. No adverse effects were identified due to the intervention.

**Conclusions:**

The Talking Time intervention is feasible and shows nonsignificant promising results with regard to the self-rated psychological HRQoL. After further adjustment, the intervention needs to be evaluated in a full trial.

**Trial registration:**

Clinical Trials: NCT02806583, June 9, 2016 (retrospectively registered).

## Background

Informal care, which means supervision, support and assistance with daily living activities, is nonprofessional care provided by people in a patient’s social environment. Usually, a spouse or a child is the informal caregiver of a person with dementia living at home [1]. Worldwide, informal caregivers remain the cornerstone for care recipients living at home [2], and half of those care recipients are people with dementia [3]. Supporting and caring for people with dementia requires time, personal engagement and day-to-day management. Due to their care responsibilities, informal caregivers of people with dementia often show higher stress levels than caregivers of physically frail elderly people [4] and have an increased risk of becoming physically and mentally ill. The care responsibility increases over the course of dementia, especially as challenging behaviors occur and cognitive abilities decline [5].

Internationally [5], the promotion of social support is a promising intervention approach that is needed due to policy requirements and the limited service offered by statutory long-term care insurance systems, which vary depending on the country-specific health care systems and the principle of subsidiarity. This principle suggests that larger social or state units should have a subsidiary function and perform only those tasks that cannot be performed by a smaller unit. In the case of the German long-term care insurance system, care is a social task that should be primarily performed by the family members of the person who is in need [6].

Available evidence suggests that social support interventions can decrease psychological and nonpsychological burdens [7], decrease social isolation and loneliness [8, 9] and protect against the occurrence of dementia [5, 10]. However, a recent review demonstrated the potential benefit of such intervention while also highlighting the generally inconsistent results. Therefore, this review recommend the performance of further high quality trials [5]. The Talking Time intervention builds on the following definition of social support by Cohen et al. [11]: “*the social resources that persons perceive to be available or that are actually provided to them by nonprofessionals in the context of both formal support groups and informal helping relationships*”.

Informal caregivers, who are permanently and continuously responsible for people with dementia, are often unable to participate in social support groups because accessibility in rural regions is poor [15–17], especially when the support is to take place outside the home [12–14]. However, new so-called remote interventions offer the possibility of contacting social support services by means of telephone calls, video calls, online networks and chat forums so that informal caregivers can keep in touch with groups offering the support needed and can thus overcome the problems of the location where they live [5].

So far, however, there are hardly any such remote interventions for social support services in Germany. In an evaluation study, Jonas and colleagues [12], identified various barriers such as a weak internet connection in rural areas, anxiety about using a computer or concerns about the internet-based intervention in general.

Telephone connections are available in almost every household in Germany and older people are usually experienced in the use of telephone technology. However, there is a lack of offers of telephone-based social support for informal caregivers of people with dementia in Germany. Likewise, no studies have been made on the effectiveness of such telephone-based services available in Germany so far.

Therefore, the purpose of the Talking Time study was to conduct the first evaluation of the feasibility and effectiveness (including possible detrimental effects) of a new intervention based on telephone-based support groups [13]. Contrary to traditional psycho-educative approaches the Talking Time intervention basically follows the principles of theme-centered interaction (TCI) according to Ruth Cohn [14]. That means that the focus of the intervention is the reciprocal exchange of experience between informal caregivers as well as joint learning. In doing so, especially the therapeutic group effect factors “universality of suffering” and “interpersonal learning” by Yalom [15] may influence their HRQoL. This is the main advantage of the TALKING TIME intervention compared to classical approaches in lecture format. Since TALKING TIME is also based on the principles of behavioral therapy [16] (problem solving), and the perspective of systemic therapy [16] (role change), the HRQoL of informal caregivers may also be influenced by these factors.

In this paper, we present the results of the recruitment, retention and effectiveness evaluation.

## Methods

### Trial design

The Talking Time study was an MRC framework phase two [17] randomized controlled trial [13, 18]. Measurements were assessed at the following two measurement points: T_0_ at the baseline and T_1_ after 3 months. The effect evaluation was based on an outcome model of the stress process of informal caregivers [19, 20]. The trial also included a continued process evaluation (will be published later) throughout the study [13]. Moreover, the trial followed the CONSORT statement [21].

### Recruitment and participants

We used the methodology previously described by Berwig and colleagues [13]. The sample consisted of informal caregivers and, if possible, the particular relative with dementia. The recruitment was based on several public relations strategies (e.g., information folder, articles in journals of health insurance companies, and announcements disseminated via relevant journals, memory clinics, Alzheimer’s disease associations and relevant websites). Those interested in participating in the study were contacted by the recruiting center by telephone or e-mail and verbally informed about the study in a telephone call. In the case of continuing interest in participating, the study information and informed consent declarations were sent to the caregivers and persons with dementia via postal mail. After the signed informed consent forms were returned to the recruiting center, the inclusion and exclusion criteria were assessed via telephone interviews.

The inclusion criteria for the informal caregivers were as follows: (1) The caregiver was living or sharing cooking facilities with the relative with dementia or providing care for a relative with dementia for at least 4 h on at least 4 days a week during the past 6 months. (2) Moreover, the informal caregivers needed access to a telephone connection to be able to participate in the intervention and the data collection procedure. (3) The relative with dementia had to have a medical dementia diagnosed based on the criteria of the International Classification of Diseases 10th Revision (ICD-10) [22]: F00.-* = Alzheimer’s disease or related disorders, F01.- = vascular dementia, or F03.- = unspecified dementia.

The exclusion criteria for informal caregivers were a lack of German language skills, an actual psychiatric diagnosis (ICD-10: F10.-*, F20.-*, F00 – F09, F05.-*, F06-*, F08, F09, or F25.-*), and a risk of suicide. Also excluded were people with dementia with the ICD diagnosis (F02.-*), except dementia due to primary Parkinson’s disease (F02.3*) and Lewy body disease (F02.8/G31.82).

The abovementioned criteria were determined via telephone and assessed by a psychologist experienced in gerontopsychiatry and psychodiagnostics. If necessary, an uncertain diagnosis was clarified with the physician who made the diagnosis by means of a release from confidentiality.

### Sample size

We used the methodology previously described by Berwig et al. [13]. The sample size calculation was based on the effect size for psychological health-related quality of life (HRQoL, primary outcome). Based on the results of one German study [23], we assumed a conservative effect size of 0.70. With consideration of the individual level randomization, a significance level of α = 0.05, a two–sided two-sample t-test, a power of 80%, an estimated dropout rate of 20% [24] and the aforementioned effect size, we computed a target sample size of 88 participants (44 in each group). The software used for the sample size calculation was G*Power [25].

### Randomization

We used the methodology previously described by Berwig and colleagues [13]. The informal caregivers were block randomized. The blocks had a length of eight, and within a block, four caregivers were allocated to the intervention group, the other four to the control group via a random permutation. These groups of four informal caregivers formed the telephone groups.

The randomization was performed by an external data manager who was not involved in the study intervention or data analysis. Only the team performing the intervention was informed about the group assignments. The statistician and researchers responsible for data collection were blinded regarding the group assignments. The inclusion criteria and baseline data of the study were assessed before the randomization of participants.

### Intervention

We used the interventions previously described by Berwig and colleagues [13].

### Intervention group

The intervention consists of four fixed components. All components are free of charge and were delivered as follows:

#### *Component 1*: telephone-based preliminary talk

Prior to the start of the support groups, the moderator conducted a preliminary telephone conversation, lasting approximately 30 min, with each informal caregiver. Information about the current care situation, information about the group process, and the rules for the group conversation were shared.

#### *Component 2*: information booklet

To support the thematic introduction of each support group meeting (component 3), each participant received an information booklet developed for the TALKING TIME study that summarized the information on the themes of self-care, access to assistance and support, communication with healthcare providers, communication with family and friends, and improving interactions with the relative with dementia (see component 3). The information booklet could be used as a workbook (e.g., for notes) during the support group meeting. The booklet also included a checklist regarding technical issues (e.g., “What is the battery status of my telephone?”) that needed to be considered prior to each group session.

#### *Component 3*: structured telephone-based support groups

Each participant had to participate in six telephone-based support group sessions. The support group sessions had a length of approximately 1 h and were scheduled to occur every 2 weeks over a three-month period. One psychologist who is experienced at working with informal caregivers of people with dementia moderated the support group session (each with four participants). At the beginning of a support group session, one of the mentioned five themes (component 2) was introduced by the moderator. After the thematic introduction, the remaining 45 to 50 min were available for a moderated exchange and discussion among the informal caregivers. At the end of a telephone call, the content of each meeting was summarized by the moderator.

#### *Component 4*: structured evaluation of each support group session

After each support group session, a structured questionnaire form was distributed to each support group participant. With this questionnaire, the informal caregivers were directed to reflect on each support group session individually. The completed questionnaire was returned to the moderator of the support group sessions. The moderator used the information to prepare for the following support group session. Moreover, after pseudonymization, the completed questionnaires were used as data sources for the process evaluation.

### Control group

The informal caregivers in the control group performed their usual individual self-organized care between T_0_ and T_1_ without any additional support related to the Talking Time trial. To enhance the study fidelity, the informal caregivers received the Talking Time intervention after the T_1_ data collection was complete.

### Outcomes

We used the outcomes previously described by Berwig et al. [13]. The primary outcome of self-rated psychological HRQoL was measured with the mental component summary (MCS) of the General Health Survey Questionnaire Short Form 12 (SF-12) [26, 27]. The SF-12 is widely used and has been shown to be feasible for telephone interviews [27].

The secondary outcomes for the informal caregivers were the self-rated physical HRQoL, social support, social conflicts, and caregiver reactions, as well as the proxy-rated challenging behavior of the care recipients with dementia.

The physical HRQoL was assessed with the second domain of the SF-12, the physical component summary (PCS). The PCS and the MCS consist of six items each with scores ranging from 0 to 100; higher scores indicate higher HRQoL. Both SF-12 component scores have shown adequate reliability and validity [28].

The perceived social support received by the informal caregivers was measured with the Perceived Social Support Caregiving instrument (PSSC, 9 items) [29, 30]. The item scores are summed to obtain a total score, which reflects the level of social support and social conflict; the scores can range from 9 to 45. Higher scores indicate a higher level of social support. The original PSSC has been demonstrated to be sufficiently reliable and valid [29]. This version was guideline-driven [31] and translated into German as a part of the Talking Time project.

The caregiver reactions were rated with the caregiver reaction scale (CRS) [32–34]. The instrument consisted of 24 items reflecting caregiver self-esteem (7 items, range: 7 to 35), lack of family support (5 items, range: 5 to 25), financial impact (3 items, range: 3 to 15), daily schedule impact (5 items, range 5 to 25), and health impact (4 items, range: 4 to 20). Based on the recommendation by Given et al. [32], we computed the subscale scores, where higher scores indicated a stronger impact. The German version of the CRS has been demonstrated to have sufficient internal consistency and structural validity [33]. In general, the secondary outcomes were assessed for the in-depth analysis of the intervention effect and possible adverse effects on the informal caregiver.

The challenging behavior of relatives with dementia was assessed to investigate possible adverse effects on the level of care received. The assessment was based on proxy ratings by informal caregivers using the Neuropsychiatric Inventory-Q (NPI-Q) [35]. This measurement makes it possible to assess the prevalence and severity of the following 12 different behaviors and psychological symptoms related to dementia: 1. delusion, 2. hallucination, 3. depression, 4. anxiety, 5. euphoria, 6. aggression, 7. apathy, 8. disinhibition, 9. irritability, 10. aberrant motor behavior, 11. sleep problems, and 12. eating disorders. The measurement results in a total score ranging from 0 to 36, with higher scores indicating more challenging behaviors. The NPI-Q has been shown to have adequate reliability and validity [35].

As control variables, the cognitive abilities, activities of daily living of the care recipient with dementia and possible social conflicts of the informal caregiver were assessed with the General Practitioner Assessment of Cognition (GPCOG, total score: 0 to 6) [36, 37], the Functional Activities Questionnaire (FAQ, total score: 0 to 30) [38, 39] and the Social Conflict Scale (SCS) [29, 30], respectively. Higher total scores indicate more impaired functions or greater social conflicts.

The sociodemographic data, e.g., age and gender of the informal caregiver and the care recipient with dementia and the care dependency level as defined by the German long-term care insurance of the person with dementia, were rated with single items. The educational level of the informal caregiver was assessed based on the procedure of the Consortium to Establish a Registry for Alzheimer’s Disease (CERAD) [40].

The data collection was performed during telephone interviews. Each participating informal caregiver received a printed TALKING TIME questionnaire with all measures and items prior to the telephone interview. Telephone interviews were initiated by members of the research team, who are registered nurses and academically qualified nursing researchers experienced in data collection procedures in dementia research. A comprehensive instruction manual regarding data collection and data handling was provided to support each interviewer.

### Statistical analysis

We used the statistical methods described by Berwig et al. [13]. The baseline characteristics of the participants were described by relative frequencies or means (± standard deviations). For the analysis of the primary and secondary outcomes, linear models were used to estimate the expected values of the dependent variables. The dependent variables were defined as the differences in the outcome measurements between the two time points (T_1_-T_0_).

Within the models, the independent variable was the study group. Based on our outcome model [41], the final model was adjusted to the baseline data of self-rated physical health, perceived social support, caregiver self-esteem, caregiver lack of family support, caregiver impact on finances score, caregiver impact on daily schedule score, caregiver impact on health score and the irritability of the people with dementia. Because of significant baseline differences between the intervention and control groups, the model was further adjusted to the T_0_ data of the FAQ score and self-rated psychological HRQoL. The usage of difference scores as the dependent variable and baseline scores as the adjusting variable is frequently applied in cases of baseline differences between groups [42, 43]. Based on missing data, the single challenging behavior irritability was used as a representative variable for challenging behaviors (as a covariate and secondary outcome). Irritability was chosen because a recent review identified irritability as the most burdensome behavior of people with dementia for informal caregivers [44].

For each group (intervention, control), the least square means (model-based estimated) and their 95% confidence intervals (CI-95%s) are presented. This analysis was repeated for all secondary outcomes using the model of the primary outcome analysis. The statistical analysis was performed using R statistical software version 3.2.4 [45]. The statistical analysis was based on the principles of intention-to-treat.

## Results

### Participant recruitment and retention

**Fig. 1:**
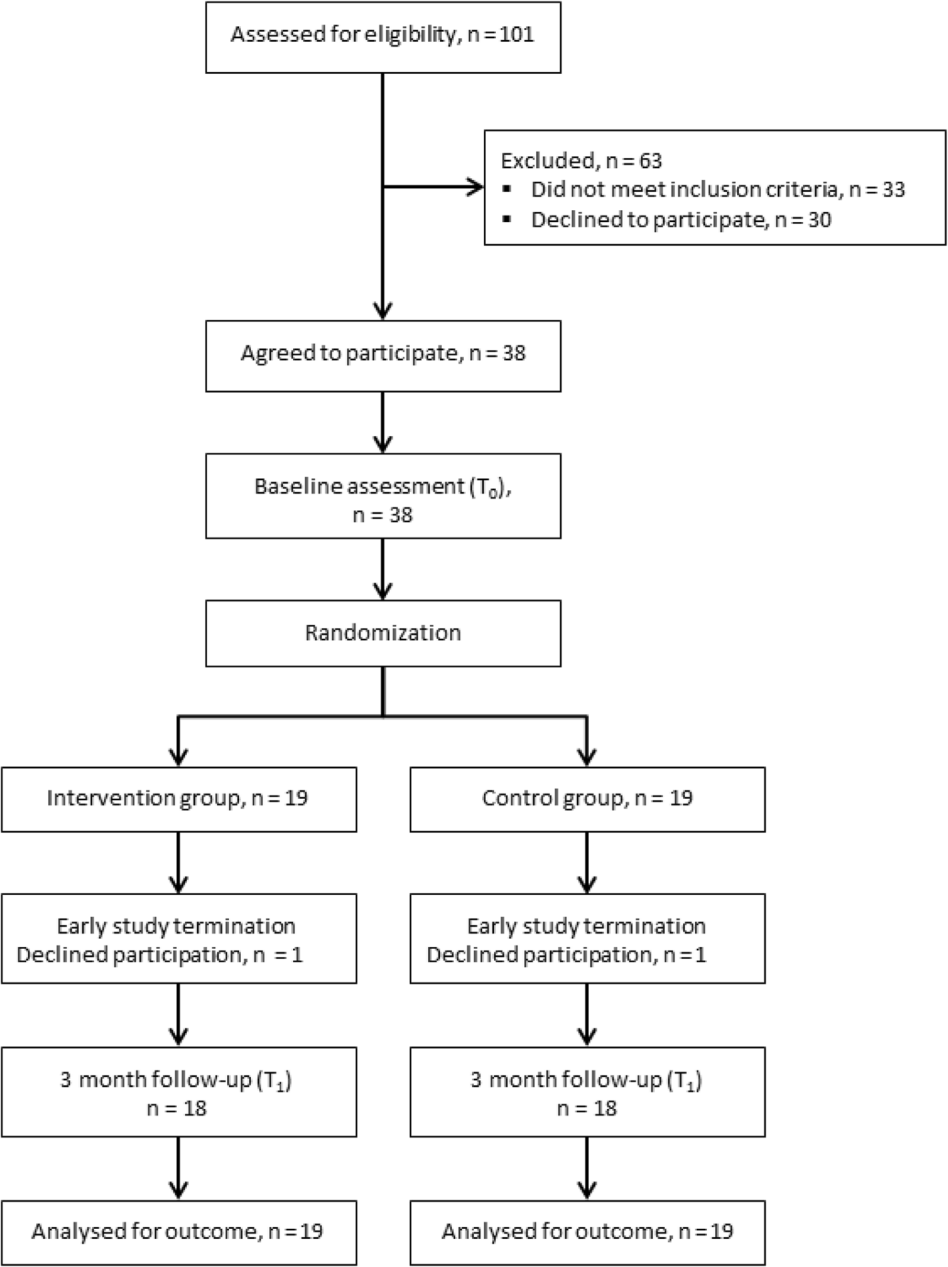
Participant flowchart

Of the 101 informal caregivers screened, 38 informal caregivers were eligible at the baseline and were incorporated into the analysis. Of these participants, 36 completed the study (Fig. 1). Among the excluded caregivers, *N* = 33 did not meet the inclusion criteria, and *N* = 30 declined to participate. Reasons for the refusal were an assumed high burden due to study participation (*N* = 4), lack of time resources for study participation (*N* = 6), different intervention content assumed (*N* = 10), declined participation by care recipient with dementia (*N =* 4) and no contact after the first call (*N =* 4). The reasons for the exclusion of potential study participants were: lack of information regarding the medical dementia diagnosis of the care recipient (*N =* 8), care recipient with a frontotemporal dementia (*N =* 6), care recipient was admitted to a nursing home (*N* = 7), the weekly time spent on care by an informal caregiver was too short (*N =* 7), potential study participants were not the relative of the person with dementia (*N* = 2), the care recipient died during the recruitment phase (*N =* 2) and an psychiatric diagnosis (*N =* 1).

Table 1 presents the baseline characteristics of the informal caregivers and their relatives with dementia. These characteristics were generally comparable between the two groups, with the exception of the self-rated psychological HRQoL for informal caregivers (*p* = 0.001) and the activities of daily living of the care recipients (*p* = 0.04). In addition, the care dependency of the people with dementia in the intervention was overall higher than that in the control group. However, the difference was not significant (*p* = 0.07).

**Table 1:** Characteristics of the informal caregivers and the people with dementia at the baseline

**T**_**0**_	**Intervention** **group**	**Control group**	**Test results** **(*p*-values)**
**Informal caregivers of people with dementia**	***N =*** **19**	***N =*** **19**	
**Sociodemographics**			
Age, years	67.4 (±8.1)	64.1 (±10.6)	0.29^a^
Women	16 (84)^d^	16 (84)^c^	0.68^a^
Number of children			0.11^b^
1	3 (18)	8 (44)	
2	8 (47)	9 (50)	
3	5 (29)	1 (6)	
5	1 (6)	0	
Level of education			0.26^b^
No educational degree (max. 7 years of education)	0 (0)	0 (0)	
School-leaving certificate	3 (16)	2 (11)	
General Certificate of Secondary Education	8 (42)	13 (68)	
Higher education entrance qualification (A-levels)	8 (42)	4 (21)	
Level of occupational education			0.73^b^
None	0 (0)	0 (0)	
Occupational training	12 (63)	13 (68)	
Academic qualification between 3 and 5 years	7 (37)	6 (32)	
Currently employed	6 (32)	8 (42)	0.5^a^
Living with person with dementia (Yes)	16 (84)	14 (78)^c^	0.6^a^
Relationship to person with dementia, spouse	12 (63)	11 (61)^c^	0.9^a^
**Outcomes**			
Self-rated psychological HRQoL score (0–100)	45.2 (±8.5)	37.0 (±10.7)^c^	**0.01**^**a**^
Self-rated physical HRQoL score (0–100)	45.5 (±10.3)	44.9 (±11.9)^c^	0.87^a^
Social support score (9–45)	28.5 (±9.3)	23.6 (±9.3)	0.12^a^
Social conflict score (3–15)	6.3 (±3.7)	6.7 (±3.6)	0.76^a^
**Caregiver Reaction**			
Caregiver self-esteem (7–35)	27.2 (±4.8)	25.1 (±5.5)^d^	0.22^a^
Lack of family support (5–25)	13.4 (±5.6)	13.2 (±4.0)	0.92^a^
Impact on finances (3–15)	7.6 (±3.3)	8.3 (±2.8)	0.49^a^
Impact on daily schedule (5–25)	18.6 (±3.6)	18.2 (±2.4)	0.71^a^
Impact on health (4–20)	10.5 (±2.6)	11.2 (±3.2)^c^	0.51^a^
**People with dementia**			
**Sociodemographics**			
Age, years	76.3 (±8.3)^c^	76.0 (±8.0)^c^	0.9^a^
Women	4 (22)^c^	8 (44)^c^	0.16^a^
Years living with dementia diagnosis	6.8 (±5.2)^c^	6.2 (±12.4)^c^	0.85^a^
Care dependency level^e^			0.07^b^
None	3 (17)	5 (28)	
1	2 (11)	7 (39)	
2	10 (56)	3 (17)	
3	3 (17)	3 (17)	
Cognition (6–0)			0.28^b^
= 0	14 (78)	11 (61)	
≥ 1	4 (22)	7 (39)	
Activities of daily living score (0–30)	26.2 (±6.2)^c^	21.6 (±7.0)^c^	0.04^a^

Data are the mean (SD) or number (%)

^a^ ANOVA

^b^ Chi-squared test

^c^ One missing

^d^ Two missing

^e^ As determined by expert raters of the medical service of the statutory long-term care

### Intervention effects

**Fig. 2:**
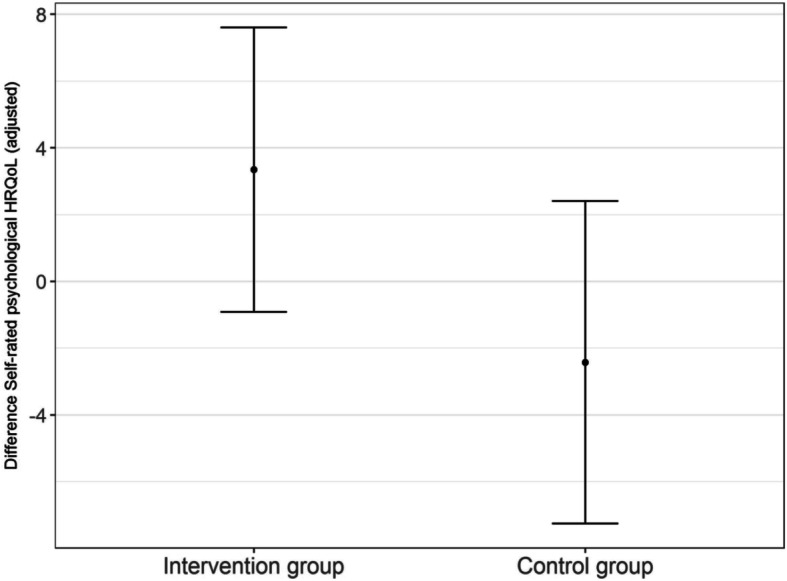
Differences in self-rated psychological HRQoL scores (estimated least square means; CI = 95%) in the intervention and control groups between T_1_ and T_0_, adjusted for covariates

The overall effect of the primary outcome, i.e., the difference in the self-rated psychological HRQoL scores between T_0_ and T_1_ as measured with the MCS of the SF-12, demonstrated a standardized effect size of 1.65, CI-95%: − 0.44 – 3.75 (covariate adjusted model in Table 2). The model without the covariate adjustment yielded similar results (Table 2: standardized effect size 0.57, CI-95%: − 1.47 – 2.60). Figure 2 illustrates the differences between the intervention and control groups.

**Table 2:** Intervention effects on the informal caregiver with regard to the primary outcome based on an intention-to-treat analysis (adjusted and not adjusted for covariates at the baseline)

**Overall** ***n =*** **38 informal caregivers**		**Difference between T**_**1**_ **and T**_**0**_
**Adjusted for covariates**^**a**^		Estimated score [95% CI]
**Self-rated psychological HRQoL score (0–100, MCS, primary outcome)**		
Intervention group (*n =* 17)		**3.3 [−0.9–7.6]**
Control group 1 (*n =* 14)		**−2.4 [− 7.3–2.4]**
Effect size^b^ Standardized effect size^c^		**5.77 [− 1.53–13.07]** **1.65[− 0.44–3.75]**
Covariates for adjustment: regression parameter [95% CI]	PCS score PSSC-Score CRS self-esteem score CRS lack of family support score CRS impact on finances score CRS impact on daily schedule score CRS impact on health score MCS score (baseline) NPI-Q irritability score FAQ score	0.1 [− 0.3–0.5] 0.1 [− 0.3–0.5] − 0.3 [− 1.2–0.7] − 0.2 [− 0.8–0.5] 0.3 [− 0.9–1.5] − 0.7 [− 2.1–0.7] − 1.2 [−3.1–0.7] − 0.5 [− 1.0–0.0] 0.9 [− 2.8–4.7] − 0.1 [− 0.8–0.6]
**Not adjusted**		
**Self-rated psychological HRQoL score (0–100, MCS, primary outcome)**		
Intervention group 2 (*n* = 18)		**1.21 [−3.03–5.44]**
Control group 1 (*n* = 17)		**− 0.48 [− 4.84–3.87]**
Effect size^b^ Standardized effect size^c^		**1.69. [− 4.39–7.77]** **0.57[− 1.47–2.60]**

^a^ Covariates: physical component score, caregiver self-esteem, caregiver lack of family support, caregiver impact on finances, caregiver impact on daily schedule, caregiver impact on health, perceived social support, irritability, functional activities, mental component score

^b^ Effect size: computed as difference in estimated scores

^c^ Standardized effect sizes: computed as difference in estimated scores divided by standard deviation

*CI-95%* confidence interval 95%, *Estimated score* model-based estimated least square means, *MCS* Mental Component Summary, *PCS* Physical Component Summary, *PSSC* Perceived Social Support Caregiving, *CRS* Caregiver Reaction Scale, *NPI-Q* Neuropsychiatric Inventory – Q, *FAQ* Functional Activities Questionnaire, *QoL-AD* Quality of Life Alzheimer’s Disease scale, *NPI-NH* Neuropsychiatric Inventory

The MCS score demonstrates a positive difference between T_0_ and T_1_ of 3.3 in the intervention group (estimated least square mean; CI-95%: − 0.9 – 7.6) compared to the control group (− 2.4, CI-95%: − 7.3 – 2.4).

For the secondary outcomes (Table 3), the adjusted differences between the intervention and control groups, were 0.12 (standardized effect size), CI-95%: CI: − 1.98 – 2.21 for self-rated physical HRQoL, 0,43, CI-95% -1.67 – 2.52 for perceived social support, − 0.31, CI-95%: − 2.41 – 1.79 for caregiver self-esteem, − 2.56, CI-95%: − 4.65 – − 0.46 for caregiver lack of family support, − 0.46, CI-95%: − 2.55 – 1.63 for caregiver impact on finances, − 0.42, CI-95%: − 2.51 – 1.68 for caregiver impact on daily schedule, 0.52, CI-95%: − 1.58 – 2.61 for caregiver impact on health and 1.22, CI-95%: − 0.87 – 3.31 for irritability of the relative with dementia.

**Table 3:** Intervention effects on secondary outcomes based on an intention-to-treat analysis (adjusted for covariates at the baseline)

**Overall** ***n*** **= 38 caregivers**		**Differences between T**_**1**_ **and T**_**0**_
**Adjusted for covariates**^**a**^		Effect Size/Estimated score [95% CI]
**Informal caregivers**		
**Self-rated physical HRQoL (0–100, PCS)**		**0.12 [− 1.98–2.21]**
Intervention group 2 (*n* = 17)		−1.7 [− 6.4–3.0]
Control group 1 (*n* = 14)		− 2.1 [− 7.4–3.2]
**Perceived Social Support Caregiving (9–45, PSSC)**		**0.43 [−1.67–2.52]**
Intervention group (*n =* 17)		3.9 [0.2–7.7]
Control group (*n =* 14)		2.6 [− 1.6–6.9]
**CRS self-esteem (7–35)**		**− 0.31 [− 2.41–1.79]**
Intervention group (*n =* 16)		− 0.3 [− 2.1–1.5]
Control group (*n =* 14)		0.1 [− 1.8–2.1]
**CRS lack of family support (5–25)**		**−2.56 [− 4.65 - -0.46]**
Intervention group (*n =* 17)		− 1.1 [− 2.2–0.1]
Control group (*n =* 14)		1.3 [0.0–2.6]
**CRS impact on finances (3–15)**		**− 0.46 [− 2.55–1.63]**
Intervention group (*n =* 17)		−0.7 [− 1.7–0.2]
Control group (*n =* 14)		−0.4 [− 1.5–0.7]
**CRS impact on daily schedule (5–25)**		**− 0.42 [− 2.51–1.68]**
Intervention group (*n =* 17)		− 0.1 [− 1.8–1.5]
Control group (*n =* 14)		0.4 [− 1.4–2.3]
**CRS impact on health score (4–20)**		**0.52 [− 1.58–2.61]**
Intervention group (*n =* 17)		0.9 [− 0.2–1.9]
Control group (*n =* 14)		0.4 [− 0.8–1.6]
**People with dementia**		
**NPI-Q irritability (0–3)**		**1.22 [− 0.87–3.31]**
Intervention group (*n =* 17)		0.2 [− 0.3–0.6]
Control group (*n =* 14)		−0.3 [− 0.7–0.2]

^a^ Covariates depending on the respective secondary outcome: physical component score, caregiver self-esteem, caregiver lack of family support, caregiver impact on finances, caregiver impact on daily schedule, caregiver impact on health, perceived social support, irritability, functional activities, mental component score

*CI 95%* confidence interval 95%, *Effect Size / Estimated score* = top row shows the standardized effect size CI and *p*-value, lower rows show the model-based estimated least square means of the intervention effect, *PCS* Physical Component Summary, *PSSC* Perceived Social Support Caregiving, *CRS* Caregiver Reaction Scale, *NPI-Q* Neuropsychiatric Inventory – Q, *FAQ* Functional Activities Questionnaire

## Discussion

The results of the Talking Time trial regarding the change in self-rated psychological HRQoL showed a promising nonsignificant difference between the intervention and control group in the expected direction (standardized effect size: 1.65 [− 0.44–3.75]). The observed nonsignificant group differences for the secondary outcomes occurred in the expected direction, with the exception of the caregiver’s self-esteem, the impact on the caregiver’s health and the irritability score for people with dementia. Overall, the differences in the secondary outcomes are small and showed no indication of possible adverse effects of the intervention.

To the best of our knowledge, there have been five previous trials regarding the effectiveness of telephone-based social support intervention for informal caregivers and their relatives with dementia. A comparison of these trials with the Talking Time study is limited because previous trials were heterogeneous in terms of design, intervention and outcome measurements [46–50]. Our study can be best compared with the design and intervention of two studies [47, 49]. Similar to the Talking Time intervention, the CONNECT intervention [49] was based on the Reach II study [50]. In the two-armed, randomized controlled trial by Martindale-Adams et al. [49], no effects were observed on patient behaviors, care burden, depression, and general well-being after twelve months. Informal caregivers participated in 15 structured telephone-based support group sessions, while others were provided with written information. The caregivers participated in sessions biweekly for the first 2 months and monthly thereafter for 1 year. Compared to the Talking Time intervention, the number of CONNECT support groups was higher, but the time period between the group sessions was longer after the first 2 months. The recruitment took place in one Veterans Affairs medical center via information leaflets placed in the center and others that were mailed to possibly relevant patients [49]. The authors mentioned no recruitment or retention difficulties and had an inclusion rate of 48% (based on the number of screened persons) and a rate of participants who were lost to follow-up of 10% [49]. In that study, inclusion criteria concerning the living situation of the informal caregivers and the care provided each week were applied [49]. In contrast to the Talking time study, this protocol did not result in recruitment challenges.

The study conducted by Winter et al. [47] was also a two-armed, randomized controlled trial evaluating the effect of telephone-based support groups for female informal caregivers after 6 months in terms of alleviating depression and the burden of care and enhancing a sense of personal gains. Similar to the Talking Time study, this trial demonstrated a nonsignificant tendency for the outcomes to change (control = usual care) in the expected direction [47]. The telephone-based groups consisted of five caregivers and a trained social worker and occurred every week. The recruitment was based on targeted mailing to adult day center users, clinical programs and newspaper ads. No inclusion criteria were applied to the living situation of the informal caregivers and the care provided per week. No detailed information regarding recruitment and retention rates was reported [47].

The aforementioned Reach II study evaluated a multicomponent intervention consisting of twelve individualized counseling sessions (nine on site and three via telephone), which were conducted over half a year. In addition, once a month for 5 months, structured telephone support group sessions took place. The trial demonstrated a significant improvement in the quality of life of caregivers and a lower prevalence of clinical depression in the intervention group compared to the control group (control = educational materials and three brief check-in calls) [50]. Unfortunately, the study provided no information about the effectiveness of their individual components, which makes a comparison with the Talking Time study difficult. This also applies to the other available studies [46, 48]. The recruitment took place at five study centers and occurred in memory disorder clinics, primary care clinics, social service agencies, physician offices, churches and community centers. In that study, information brochures were used. Moreover, public service announcements on radio stations, newspaper articles, television, targeted newsletters and community presentations were used [50]. Based on this extensive recruitment procedure, the recruitment rate was 65%, and no challenges concerning recruitment were reported. The inclusion criteria regarding the living situation of informal caregivers and the care provided per week were the same for the REACH II study [50] and the Talking Time trial. The rate of participants who were lost to follow-up after 6 months was 9% [50].

This comparison between recruitment and retention procedures and rates reveals the need for a more advanced recruitment procedure for an MRC framework phase III trial investigating the Talking Time intervention. First, we recommend the inclusion of more recruitment centers and financial funding for public relations strategies targeting informal caregivers and their relatives with dementia who are living at home. Second, if possible, informal caregivers who participated in this study should be integrated as testimonials for the recruitment of study participants in a future trial.

Third, the participation of informal caregivers as part of the planning team for a future trial and especially for the planning of the recruitment approach is recommended.

Fourth, recruitment materials or media for a future trial, such as a trial website or a folder, should include a detailed description of the intervention and the inclusion and exclusion criteria in plain language. The fact that the intervention was group-based seems to have had no effect on recruitment.

### Strengths and limitations

The main strengths of our trial are that it is the first evaluation of the Talking Time intervention based on a rigorous experimental design. The applied study design and intervention resulted in a small dropout rate of 5% of the study participants. The outcome model of the Talking Time study is based on the models of informal caregivers’ stress processes [13]. Our results give no indication that one of the secondary outcomes is preferable as a primary outcome in future studies. The application of outcomes such as depression [47, 49] seems to not adequately reflect the components of the Talking Time intervention.

Our methodological approach was accompanied by various limitations. First, the preplanned sample size was not achieved (target sample size: *n* = 88; realized sample size: *n* = 38). Thus, this study has low statistical power and type II error is possible. A new sample size calculation based on the planned values (effect size = 0.7, α = 0.05) and the sample size of *N* = 38 demonstrated a power of = 55.56%. The possible reasons for this unsatisfactory sample size included a restricted recruitment time and rigorous inclusion criteria (e.g., providing care for a relative with dementia for at least 4 hours on least 4 days each week during the past 6 months), the use of only one recruitment center and the lack of financial funding for public relation strategies to inform informal caregivers and their relatives about the opportunity to participate in the study.

Second, our primary outcomes of the self-rated psychological HRQoL and the FAQ score showed significant baseline differences between groups. The baseline difference in the self-rated psychological HRQoL scores, with higher scores in the intervention group and lower scores in the control group, may have led to an underestimation of the effect size. All participants in the intervention and control groups knew that they were part of a trial (Hawthorne effect). This knowledge may have influenced the participants in both groups. However, this does not result in a confounded comparability of the group results.

Third, our study design did not allow analysis of the long-term effects of the Talking Time intervention. Another trial should be conducted to investigate the long-term effects after 6 months of the intervention.

Fourth, apart from the Talking Time intervention, we have not assessed additional types (e.g. respite care) of informal caregiver support during the intervention phase. For a future trial we recommend that this data are collected during the intervention period.

Fifth, information regarding individual actions of each informal caregiver to participate in the telephone-based social support groups and the consequences of the Talking Time intervention for the respective care arrangement can only be answered after the analysis of the process evaluation data.

## Conclusion

The results of our trial identified a promising but not statistically significant change in self-rated psychological HRQoL scores after intervention. The recruitment process was more difficult than expected. The comparison with previous trials leads to the assumption that there is a need for an advanced recruitment procedure including several recruitment centers and financial funding for public relations strategies targeting informal caregivers and their relatives with dementia living at home. Moreover, less stringent inclusion criteria regarding caregivers may be more useful. For example, it may be sufficient for inclusion if a person who cares for a person with dementia is the primary caregiver and responsible for the stability of care. In addition, recruitment materials, that include a detailed description of the intervention and the inclusion and exclusion criteria in plain language, will be helpful to make participant recruitment successful. In addition, informal caregivers themselves should be part of the planning team of a future trial and especially with regard to the recruitment strategy.

Finally, the results of the study and the results of our process evaluation (in preparation) will provide insight into the further development of the intervention. The process evaluation will give additional information regarding fidelity, dosage and context. The fidelity results will provide information on the satisfaction of the study participants with the intervention components and on the extent to which the intervention meets the needs of informal caregivers of people with dementia. In general, our study protocol appears to be feasible, with the exception of the recruitment procedure.

For comparison reasons, a harmonization of study designs and outcomes should be sought for future trials investigating telephone-based social support interventions (e.g., follow-up assessment 6 months after the intervention).

## Data Availability

All of the data necessary for a meta-analysis are contained within the manuscript and its supplementary files. The datasets used and/or analyzed during the current study are available from the corresponding author on reasonable request.

## Abbreviations

CERAD: Consortium to Establish a Registry for Alzheimer’s Disease
CRS: Caregiver reaction scale
FAQ: Functional Activities Questionnaire
GPCOG: General Practitioner Assessment of Cognition
ICD-10: International Classification of Diseases 10th Revision
HRQoL: Health-related quality of life
MCS: Mental component summary
NPI-Q: Neuropsychiatric Inventory-Q
PCS: Physical component summary
PSSC: Perceived Social Support Caregiving instrument
PwD: People with dementia
SCS: Social Conflict Scale
SF-12: General Health Survey Questionnaire Short Form 12
TCI: Theme-centered interaction
